# Cobimetinib‐induced “dropped head syndrome” and subsequent disease management in an Erdheim‐Chester patient

**DOI:** 10.1002/ccr3.2297

**Published:** 2019-09-10

**Authors:** Amber C. King, Eli L. Diamond, Jennifer S. Orozco, Hannah R. Morse, Linda L. Ouyang, Heiko Schöder, Raajit K. Rampal

**Affiliations:** ^1^ Department of Pharmacy Memorial Sloan Kettering Cancer Center New York New York; ^2^ Department of Neurology Memorial Sloan Kettering Cancer Center New York New York; ^3^ Department of Nursing Memorial Sloan Kettering Cancer Center New York New York; ^4^ Department of Radiology Memorial Sloan Kettering Cancer Center New York New York; ^5^ Department of Leukemia Memorial Sloan Kettering Cancer Center New York New York

**Keywords:** Erdheim‐Chester disease, hematology, neurology, pharmacology

## Abstract

Our rechallenge of cobimetinib in an Erdheim‐Chester Disease (ECD) patient for the rare adverse effect, “dropped head syndrome,” with a previously unexplored cobimetinib regimen was successful. Similar to other experiences with targeted agents in ECD, dosing of cobimetinib may vary to mitigate toxicity without impairing efficacy.

## 1 INTRODUCTION

Erdheim‐Chester Disease (ECD) is a rare non‐Langerhans cell histiocytic disorder, characterized by sequelae resulting from histiocytic infiltration of skeletal and extraskeletal tissues. Active surveillance may be employed for cases of mild or asymptomatic disease until end‐organ dysfunction or symptoms develop.

Considering the rare incidence and heterogenous presentation of symptomatic disease, there is no consensus on treatment approach. The goals of therapy are minimization of toxicity, abrogation of symptoms, and prevention of end‐organ dysfunction.

The clinical presentation of ECD varies depending upon site(s) of involvement. Most patients with ECD will have skeletal involvement at the time of diagnosis and at least one additional nonskeletal site of involvement. Notably, cardiovascular involvement with ECD has been reported in many cases, lending to significant morbidity and mortality.^1^, ^2^

Many patients with ECD harbor the *BRAF* V600E mutation. The *BRAF* inhibitor, vemurafenib, has demonstrated efficacy in this population. FDA approval was granted based on a study of 22 patients with *BRAF* V600 mutation‐positive ECD; most patients had previously treated disease. Dose reductions for arthralgias, maculopapular rash, fatigue, and other toxicities were common. Vemurafenib resulted in a 55% overall response rate, despite an appreciable rate of dose attenuation.^3^, ^4^

For those without *BRAF* V600E mutations, additional therapies for relapsed and/or refractory disease are needed. The recent implication of the *MAPK* and *ERK* pathways suggests *MEK* inhibitors may be of clinical benefit.^5^

Cobimetinib is a reversible inhibitor of *MEK*1 and *MEK*2, currently FDA approved for unresectable or metastatic *BRAF* V600E/K mutated melanoma in combination with vemurafenib. Common toxicities associated with single‐agent cobimetinib include nonacneiform rash, ocular disorders, and creatine phosphokinase elevations (CPK). There is a risk of cardiomyopathy that is increased with combination vemurafenib and cobimetinib that exceeds previously reported incidence with vemurafenib monotherapy.^6^, ^7^

A case series evaluated cobimetinib in histologically proven *BRAF* V600E wild‐type ECD disease in patients with renal and cardiac involvement. Radiographic and laboratory remissions were achieved, with no major toxicities reported.^8^

Here‐in, we describe our experience with a *BRAF* wild type, *KRAS* G12S‐positive ECD patient treated with cobimetinib that developed “dropped head” syndrome (DHS).

## 2 CASE REPORT

**Figure 1:**
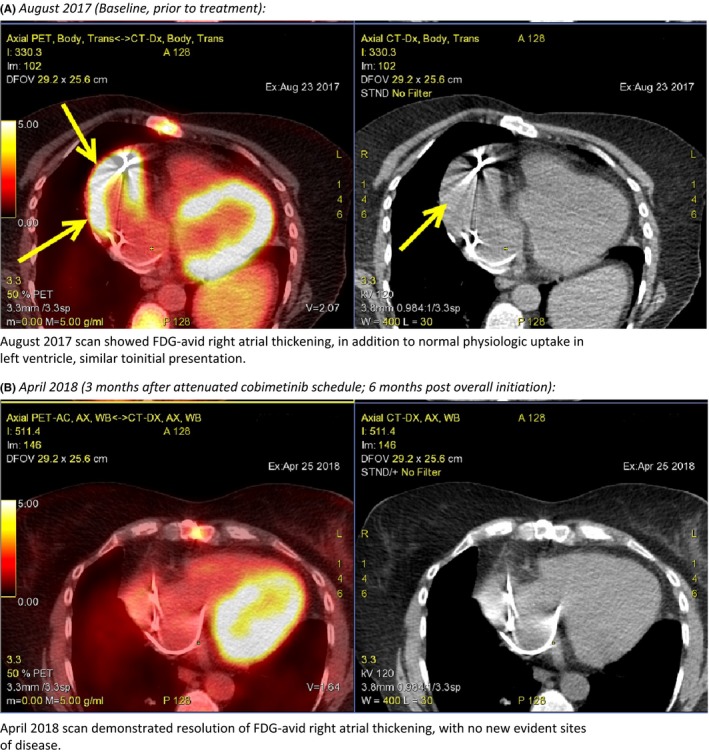
Radiographic demonstration of disease response (PET CT). A, August 2017 (Baseline, prior to treatment): August 2017 scan showed FDG‐avid right atrial thickening, in addition to normal physiologic uptake in left ventricle, similar to initial presentation. B, April 2018 (3 mo after attenuated cobimetinib schedule; 6 months post overall initiation): April 2018 scan demonstrated resolution of FDG‐avid right atrial thickening, with no new evident sites of disease

A 71‐year‐old female with a history of *BRAF* wild type, *KRAS* mutated ECD in 2013 with right atrial and superior vena cava (SVC) involvement was initiated on PEG‐interferon, titrated to 135 micrograms, for symptomatic disease. Recent pacemaker placement precluded further surveillance via cardiac MRI (magnetic resonance imaging), thus PET (positron emission tomography) scans were utilized for further disease assessment. She achieved a partial response (PR) based on PET/CT after 12 doses and was maintained on therapy for years. In August 2017, PET CT (computed tomography) demonstrated increased cardiac SUV, suggestive of disease progression (Figure 1A). After increased subjective symptoms and failure to enroll on clinical trial, off‐label cobimetinib 40 mg/d for 3 weeks on and 1 week off was initiated. Cobimetinib was initiated at 40 mg secondary to CYP3A4 mediated drug‐drug interaction with amiodarone therapy. Her baseline CPK (creatine phosphokinase) was at the lower limit normal (24 U/L).

At cycle 1 day 15, the patient noted significant neck stiffness but denied trauma and/or weakness during routine follow up. Pertinent laboratory studies were stable or within normal limits. At cycle 1 day 28, the patient endorsed left‐sided neck pain, limiting ability to lift the head. A CT Neck performed locally reported only moderate left foraminal C4‐C5 narrowing and mild degenerative changes. Laboratory studies were unremarkable. Prior to cycle 2, she reported increased neck pain and reduced mobility. Neck pain resolved after a short course of methylprednisolone, facilitating the initiation of cycle 2. On cycle 2 day 8, she endorsed persistent neck pain, with a new elevation in CPK to 150 U/L (six times the baseline value). The decision was made to hold cobimetinib. During interval follow up, 21 days after drug cessation, there was subjective improvement in neck mobility. CPK down‐trended to 44 U/L, approximately twice the baseline value.

**Figure 2:**
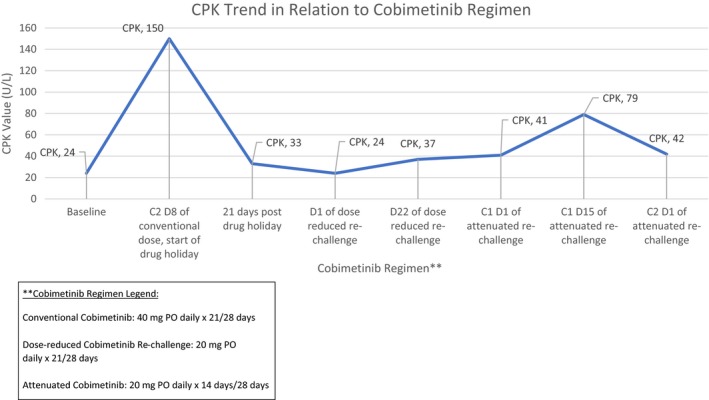
CPK trend in relation to cobimetinib regimen. **Cobimetinib Regimen: Conventional cobimetinib: 40 mg PO daily × 21/28 d. Dose‐reduced cobimetinib re‐challenge: 20 mg PO daily × 21/28 d. Attenuated cobimetinib: 20 mg PO daily × 14/28 d

At day 50 post cessation, cobimetinib rechallenge was instated at 20 mg daily for 3 weeks on and 1 week off. On day 8 of the rechallenge, she reported continued improvement of neck strength and mobility, denying any resurgence of adverse events. All laboratory findings were within normal limits, including a CPK of 24 U/L (return to baseline value). On day 22, the patient reported a slight worsening of head drop, still with full range of motion, but now requiring focused effort to achieve neck mobility (CPK increased to 37 U/L). Our patient subsequently reported improvement during her off week. Taking the aforementioned events into consideration, we explored an attenuated dosing strategy of cobimetinib 20 mg/d, 2 weeks on and 2 weeks off in an effort to balance efficacy and toxicity. At follow up on cycle 1 day 15 of the attenuated rechallenge, she reported overall improvement in head drop and no new focal complaints, despite elevated CPK of 79 U/L. Prior to initiation of cycle 2, CPK decreased to approximately twice the baseline value with continued improvement of symptoms (Figure 2). The most recent PET CT demonstrated resolution of cardiac FDG avidity with no new lesions of concern. (Figure 1B). Our patient continues the attenuated rechallenge schedule of cobimetinib, noting minor and intermittent resurgence of symptoms approximately day 4‐6 of each cycle, with complete resolution by day 28.

## 3 DISCUSSION

Dropped head syndrome is characterized by muscle weakness that often precludes the ability to raise the head. The most common causes are motor neuron pathologies, such as Parkinson's disease and necrotizing myopathies, including focal necrotizing myopathy.^9^

Our report is clinically discordant from CPK elevations described in clinical trials with *MEK* inhibitors, as these cases were mostly asymptomatic. There were no reports of rhabdomyolysis or symptomatic CPK elevations.^6^, ^7^

This case also differs from previous reports of DHS in the metastatic melanoma population, with all cases demonstrating radiographic changes in affected muscles and marked CPK elevation.^10^, ^11^ The patient's symptoms occurred at a lower peak CPK (150 U/L). Our patient's baseline CPK was below lower limit of normal, indicating incremental changes may also correlate with symptoms.

*ERK* activity is implicated in lipid metabolism. Recent data suggest fatty acid uptake and oxidation are *ERK*1/2 dependent in vivo. *MEK* inhibition may mitigate upstream signaling, ultimately impairing fatty acid uptake, possibly lending to muscle fatigue/weakness.^12^

## 4 CONCLUSION

We describe our approach of the successful rechallenge of cobimetinib, with a previously unexplored regimen. The success of this approach may be attributed to the allowance of adequate drug wash‐out, which would be predicted to reach clinically insignificant concentrations approximately 10 days after cessation.^7^ Additionally, muscle rehabilitation in the absence of drug insult likely contributed to improvement in symptoms and resilience during subsequent cycles. Most notably, this approach did not impact treatment efficacy, with our patient experiencing a complete resolution in previously noted cardiac disease (Figure 1B). Our experience suggests the optimal dosing of cobimetinib, similar to reports of other targeted therapies in ECD, may vary to mitigate toxicity without impairing efficacy.^3^ Practitioners should be aware of this potential adverse effect and consider periodic assessment of symptoms, including a baseline neurologic evaluation prior to initiation of therapy in at‐risk patients.

## CONFLICT OF INTEREST

A. C. K.: (advisory board) for Genentech, H.S.: (consultancy) Aileron Therapeutics, E.L.D.: is supported by the Erdheim‐Chester Global Alliance and the Frame Fund, and this work is also funded in part through the NIH/NCI Cancer Center Support Grant P30 CA008748, R.K.R.: has received consulting fees from Incyte corporation, Celgene corporation, Agios Pharmaceuticals, Apexx oncology, BeyondSpring, Partner Therapeutics, and Jazz Pharmaceuticals, and has received research funding from Constellation pharmaceuticals, Incyte corporation, and Stemline Therapeutics. The remaining authors declare no competing financial interests.

## AUTHOR CONTRIBUTIONS

ACK: designed and established the study, collected and analyzed data, and wrote the manuscript; RKR: designed and established the study, supervised the research, and edited the manuscript; ELD, JSO, HRM, LLM, HS: reviewed and approved the final version of the manuscript.
